# Changing Characteristics of Rotavirus Diarrhea in Children Younger than Five Years in Urban Bangladesh

**DOI:** 10.1371/journal.pone.0105978

**Published:** 2014-08-29

**Authors:** Mohammad Habibur Rahman Sarker, Sumon Kumar Das, Shahnawaz Ahmed, Farzana Ferdous, Jui Das, Fahmida Dil Farzana, Abu S. M. S. B. Shahid, K. M. Shahunja, Mokibul Hassan Afrad, Mohammad Abdul Malek, Mohammod Jobayer Chisti, Pradip Kumar Bardhan, Md Iqbal Hossain, Abdullah Al Mamun, Abu S. G. Faruque

**Affiliations:** 1 International Centre for Diarrhoeal Disease Research (icddr,b), Dhaka, Bangladesh; 2 School of Population Health, The University of Queensland, Brisbane, Australia; National Institutes of Health, United States of America

## Abstract

**Background:**

Childhood rotavirus diarrhea is still one of the major public health challenges. The present study aimed to determine changing characteristics of rotavirus diarrhea in under-5 children at two periods of time.

**Methods:**

We enrolled 5,357 under-5 children with rotavirus positive in two different time periods; i) 1993-1997 (n = 2,493), and ii) 2008–2012 (n = 2,864) considering beginning and ending of two decades. These children were enrolled in the urban Dhaka Hospital of icddr,b.

**Results:**

Overall, proportion of rotavirus was about 25% in 1993–97, which was 42% in 2008–12 (68% rise; p<0.001). Significant higher proportion of children were stunted [38% vs. 22%; aOR-1.33 (95% CI-1.09-1.62)], had vomiting [87% vs. 74%; aOR-2.58 (95% CI-2.02-3.28)], fever [10% vs. 8%; aOR-1.31 (95% CI-0.96-1.78)], family members >5 [38% vs. 35%; aOR-1.32 (95% CI-1.10-1.58)] required more intravenous fluid [9% vs. 3%; aOR-4.93 (95% CI-3.19-7.63)], had higher co-infection with *Shigella* [3% vs. 1%; aOR-3.36 (95% CI-1.61-7.03)], *Vibrio cholerae* [4% vs. 1%; aOR-3.70 (95% CI-2.12-6.46)]; and ETEC [13% vs. 7%; aOR-2.21 (95% CI-1.65-2.97)]; however, significantly lower proportion of them used sanitary toilets [54% vs. 78%; aOR-0.66 (95% CI-0.54-0.80)], boiled drinking water [16% vs. 38%; aOR-0.60 (95% CI-0.48-0.74)], used antimicrobial at home [63% vs. 82%; aOR-0.56 (95% CI-0.46-0.69)] and had some or severe dehydration [18% vs. 34%; aOR-0.15 (95% CI-0.12-0.20)] in 1^st^ observation period compared to that of 2^nd^.

**Conclusion:**

Proportion of episodes of under-5 rotavirus diarrhea increased over the period. Concomitant changes in host, socio-demographic and clinical characteristics, and co-infections were also observed. Thus, vaccination campaign which is prevailing in private sector should also be introduced in public sector.

## Introduction

Childhood diarrhea is still contributing as the second leading cause of under-5 mortality and morbidity [1]. Rotavirus is one of the major pathogens responsible for yearly 111 million episodes of diarrhea and over 400,000 deaths among under-5 children globally [2]. It is also a leading cause of infantile gastroenteritis accounting 20% of diarrhea-associated deaths [3]. Literatures also documented rotavirus diarrhea attributing an estimated 39% of hospitalized under-5 childhood deaths and majority of them happening in resource poor settings of low and middle income countries [4], [5].

Mass campaigns for home based management with oral rehydration salt solution (ORS), and use of zinc as adjunct therapy [6], [7] in addition to vaccination against rotavirus not only reduce the diarrheal episodes but also decrease mortality, hospital stay and help improved case management [8]–[10]. Several host and socio-demographic factors have been thought to contribute high global prevalence of childhood rotavirus diarrhea such as personal and family hygiene practices as documented elsewhere [11], [12]. However, changing trend of all these indicators have not been well documented in medical literatures. The Diarrheal Disease Surveillance System (DDSS) of International Centre for Diarrhoeal Diseases Research, Bangladesh (icddr,b) systemically enrolls individuals with diarrhea irrespective of age, sex, socio-economic status and disease severity. For us, its data base paved the way to study changing characteristics of rotavirus diarrhea among under-5 urban children between two 5-year time periods of last two decades such as 1993-1997 and 2008-2012.

## Materials and Methods

### Study site

Dhaka Hospital of icddr,b; established in 1962 in Dhaka, the capital city of Bangladesh, provides care and treatment to people with diarrheal diseases mostly from urban and peri-urban areas. Over 140,000 diarrheal patients receive such services each year. A DDSS has been operating since 1979 [13], [14], which systematically sampled 4% of all patients up to 1995, however currently samples 2% since 1996. DDSS collects information on the clinical, epidemiological and demographic characteristics, feeding practices, particularly of infants and young children irrespective of age, sex and disease severity or socioeconomic status by administering a structured questionnaire. A trained research assistant interviews either the patient himself or the caregiver in case of young children following the questionnaire (detail was described elsewhere [13], [14]).

### Study population

**Figure 1:**
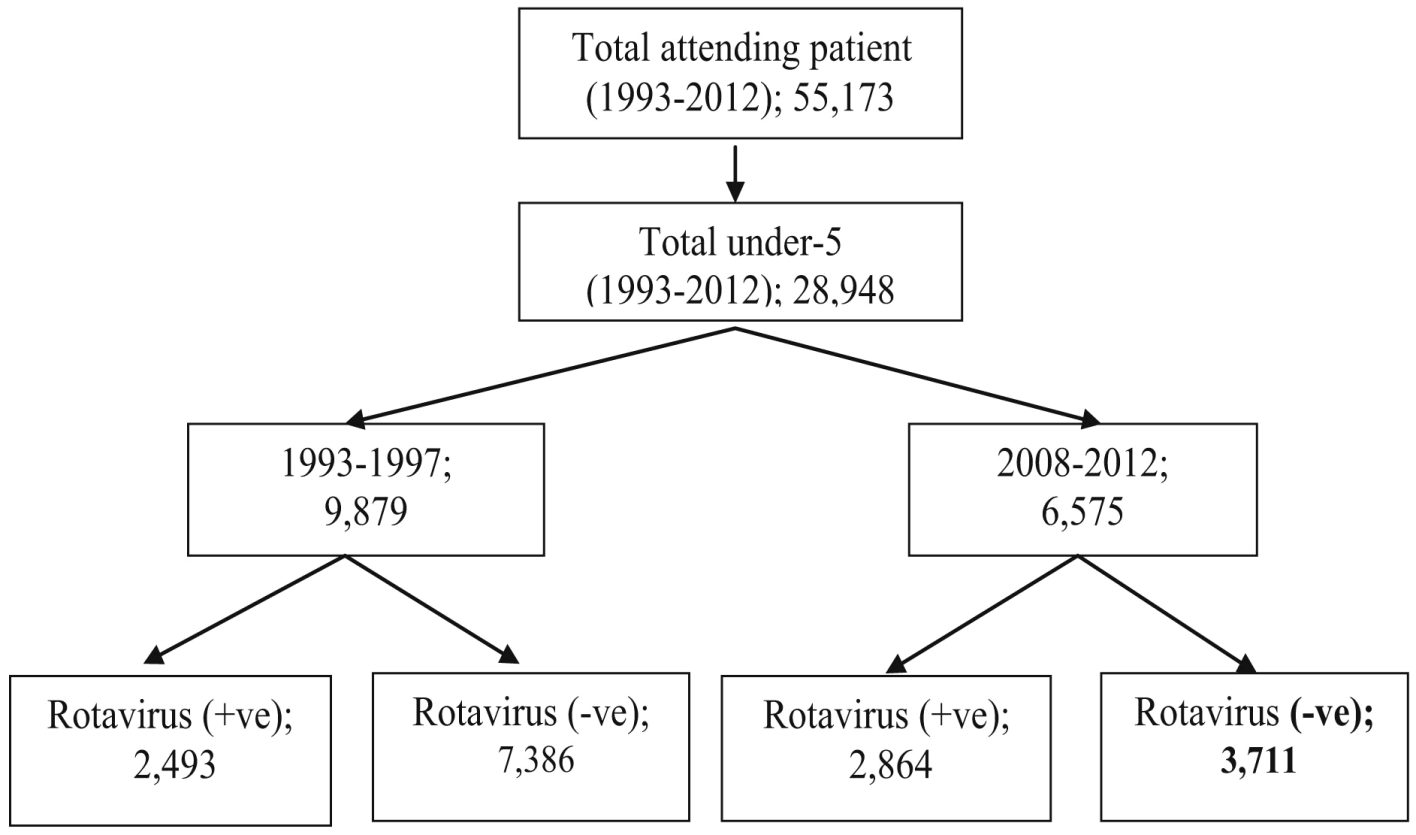
Sample framing.

A total of 55,173 patients were enrolled into the DDSS from 1993–2012 who were admitted in Dhaka Hospital of icddr,b. Of them 28,948 (52%) were under-5 children. They were divided into two time periods (i) 1993–1997 (9,879), and (ii) 2008–2012 (6,575) to determine the changing characteristics of these young childhood population between the starting and ending of two different five-year periods of last two decades. A total of 5,357 under-5 children presented with rotavirus diarrhea; of them, 2,493 (25%) under-5 children during 1993–97, and 2,864 (43%) during 2008–2012 constituted analyzable study sample size (Figure 1).

### Specimen collection and laboratory procedure

A fresh stool specimen is routinely collected from the surveillance patients for screening of common diarrheal pathogen such as rotavirus, *Shigella* spp., *Vibrio cholerae*, Enterotoxigenic *Escherichia coli* (ETEC) following standard laboratory method [15], [16].

### Definition

Diarrhea was defined as passage of 3 or more abnormally loose or liquid stools per day, or more frequently than normal for the individual. Well nourished children were defined as individual with weight-for-age z-score (WAZ), height-for-age z-score (HAZ), and weight-for-height z-score (WHZ) ≥−2.00 SD. However, malnutrition was defined as children with any of the indicators of malnutrition WAZ, HAZ, WHZ <−2.00 SD.

### Ethics

The DDSS of icddr,b has been approved by the Research Review Committee and the Ethical Review Committee of icddr,b which currently enrolls every 50^th^ patient attending for the treatment to the Dhaka Hospital of icddr,b. At the time of enrollment, verbal consent was taken from the caregivers or guardians on behalf of the patients. The information was stored in the hospital database and used for conducting researches. Although the DDSS of icddr,b is a scheduled activity on the hospital patients, and performed after taking verbal consent from the parents or guardians of the patients following the hospital policy. This verbal consent was documented by keeping a check mark in the questionnaire which was again shown to the patient or the parents. Parents or guardians were assured about the non-disclosure of information collected from them, and were also informed about the use of data for analysis and using the results for improving patient care activities as well as publication without disclosing the name or identity of their children. ERC was satisfied with the voluntary participation, maintenance of the rights of the participants and confidential handling of personal information by the hospital physicians and has approved this consent procedure.

### Data analysis

Statistical Package for Social Sciences (SPSS), Windows (Version 15.2; CPSS Inc) and Epi Info (Version 6.0, USD, Stone Mountain, GA, USA) were used for data analysis. Cumulative comparisons were done for all variables between two observation periods (i) 1993–1997, and (ii) 2008–2012. Chi-square test was performed for estimation of odds ratios (OR) and their 95% confidence intervals (CI) to determine the strength of association considering a probability of <0.05 (type I error) as statistically significant. Chi-square for trend was also performed to determine whether the changing trend of rotavirus diarrhea children over the period occurred at statistically significant level. Finally, logistic regression analysis was done to determine independent association of all the explainable variables with the (1993–1997 = 1, 2008–2012 = 0) outcome variable of interest. Additionally, for each observation point distribution of characteristics were also observed between rotavirus positive children with rotavirus negative children by employing both univariate and multivariate analysis.

## Results

**Figure 2:**
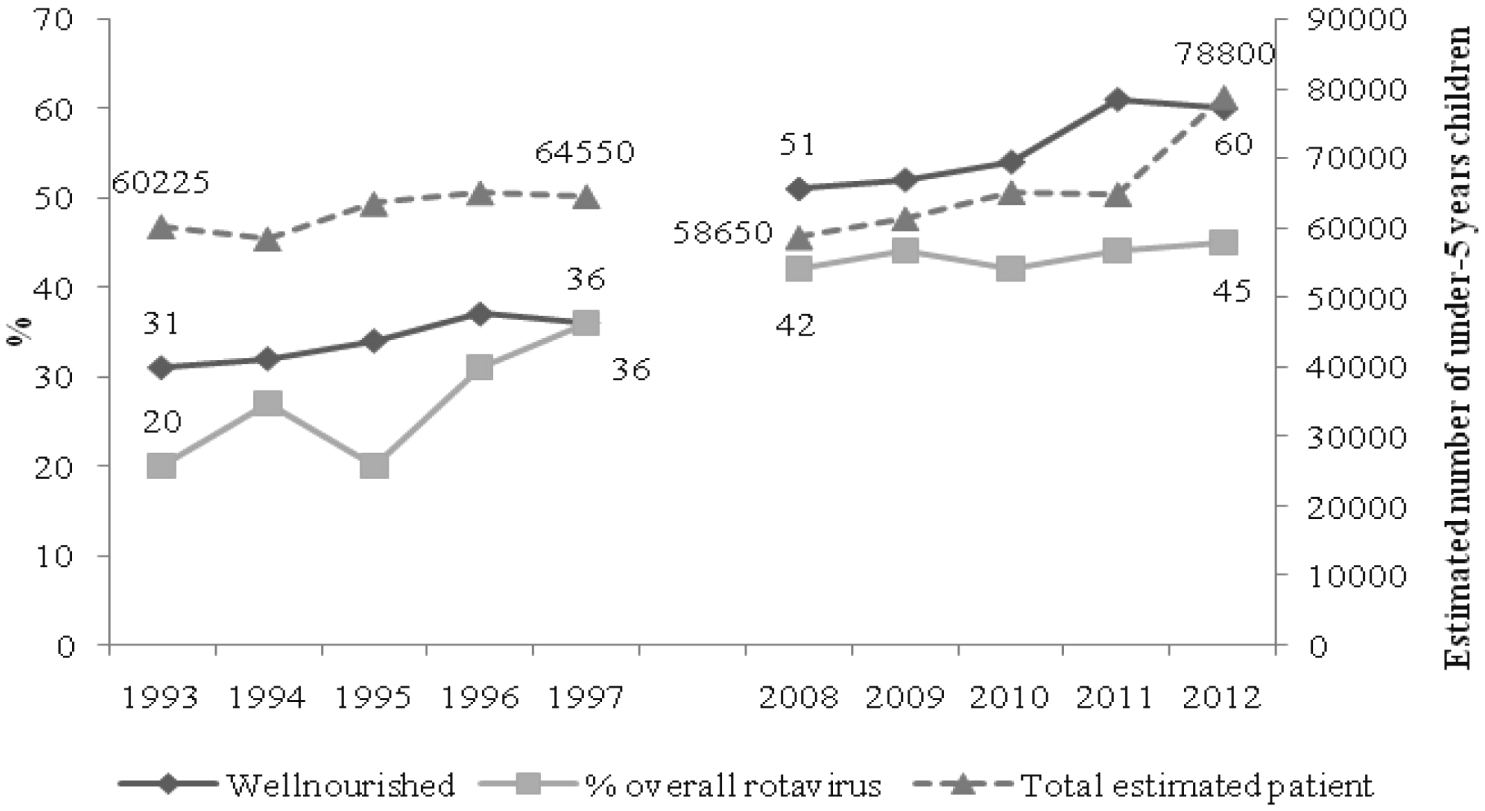
Overall and nutrition specific distribution of rotavirus diarrhea among under-5 children in two observation periods (1993–1997 and 2008–2012).

Overall isolation rate of rotavirus was 20% in 1993 which increased to more than double (45%) in 2012. Among the patients of rotavirus diarrhea, proportion of malnourished children was 57% in 1993, which decreased to 37% (35% reduction) in 2012. On the other hand, among well nourished under-5 children, it increased from 43% to 63% from 1993 to 2012 (46% increase) On the other hand, over the study period, total estimated number of under-5 children attending in the hospital was also increased from 60225 to 78800 (Figure 2). Similar increase in proportion of well nourished children was also observed within the study period (Figure 2).

Among the rotavirus diarrhea children, significant increase in the proportion of maternal literacy and the period of diarrheal hospitalization more than 24 hours was noted in 2^nd^ observation period than that of 1^st^ observation period (Table 1). Similarly, sanitary toilet use, boiled water drink, antimicrobial use at home, stool frequency more than 10 times in last 24 hours; and presentation with some or severe dehydration were also increased between the periods. However, family size (>5), fever, abdominal pain, vomiting, intravenous fluid use, hospital stay (≥24 hours) and malnutrition (underweight, stunting, and wasting) significantly decreased from 1^st^ to 2^nd^ observation period (Table 1). Considering co-pathogens such as *Vibrio cholerae*, *Shigella* spp. and ETEC significantly decreased from 1^st^ period of observation to 2^nd^ period (Table 1).

**Table 1:** Changing socio-demographic, clinical and host characteristics, and co-infection among under-5 children with rotavirus diarrhea in two observation periods (1993–1997, and 2008–2012).

Variables	1993-1997; n = 2,493 (%)	2008-2012; n = 2,864 (%)	OR (95% CI)	aOR (95% CI)
Male	1538 (62)	1813 (63)	0.93 (0.83–1.04)	-
Maternal literacy	1312 (53)	2387 (83)	0.22 (0.20–0.25)*	0.31 (0.26–0.38)*
Use sanitary latrine	1348 (54)	2228 (78)	0.34 (0.30–0.38)*	0.66 (0.54–0.80)*
Family size (>5 members)	954 (38)	1018 (35)	1.12 (1.00–1.26)*	1.32 (1.10–1.58)*
Slum residence	283 (11)	107 (4)	3.30 (2.61–4.18)*	1.32 (0.92–1.89)
Boiled drinking water	402 (16)	1078 (38)	0.32 (0.28–0.36)*	0.60 (0.48–0.74)*
Use of antimicrobial at home	1558 (63)	2339 (82)	0.37 (0.33–0.42)*	0.56 (0.46–0.69)*
History of measles within 6 months	225 (9)	73 (2)	3.79 (2.87–5.01)*	1.76 (1.12–2.78)*
Vomiting	2164 (87)	2125 (74)	2.29 (1.98–2.65)*	2.58 (2.02–3.28)*
Fever	262 (10)	233 (8)	1.33 (1.10-1.60)*	1.31 (0.96–1.78)
Abdominal pain	491 (20)	463 (16)	1.27 (1.10–1.47)*	2.06 (1.66–2.55)*
Duration of diarrhea (>24 hours)	1883 (76)	2227 (78)	0.88 (0.78–1.00)	-
Number of stool in last 24 hours (>10 times)	1058 (42)	1525 (53)	0.65 (0.58–0.72)*	0.67 (0.56–0.80)*
Watery stool	2408 (97)	2796 (98)	0.69 (0.49–0.96)*	1.24 (0.71–2.17)
Some or severe dehydration	457 (18)	985 (34)	0.43 (0.38–0.49)*	0.15 (0.12–0.20)*
Use of intravenous fluid	216 (9)	79 (3)	3.33 (2.54–4.37)*	4.93 (3.19–7.63)*
Hospital stay ≥24 hours	1048 (43)	1196 (42)	1.01 (0.91–1.13)	-
Underweight (WAZ<−2)	1110 (45)	747 (26)	2.26 (2.01–2.54)*	-
Stunted (HAZ<−2)	940 (38)	626 (22)	2.15 (1.90–2.43)*	1.33 (1.09–1.62)*
Wasted (WHZ<−2)	611 (25)	530 (19)	1.42 (1.24–1.62)*	-
*Vibrio cholerae*	94 (4)	32 (1)	3.47 (2.28–5.31)*	3.70 (2.12–6.46)*
*Shigella*	76 (3)	21 (1)	4.26 (2.56–7.14)*	3.36 (1.61–7.03)*
ETEC	112 (13)	193 (7)	2.06 (1.60–2.65)*	2.21 (1.65–2.97)*

*p<0.05; aOR-Adjusted Odds ratio;

ETEC- Enterotoxigenic *Escherichia coli.*

In multivariate analysis, significant changes were also observed between two observation periods. The variables associated were maternal literacy, use of sanitary toilets, family members (>5), drinking of boiled water, use of antimicrobial at home, vomiting, abdominal pain, some or severe dehydration, use of intravenous fluid, number of stool in last 24 hours (>10 times), stunting, *Vibrio cholerae*, *Shigella* spp. and ETEC after adjusting for other variables which significantly differed between the two observation periods (Table 1). Similar findings were observed when other indicators of malnutrition such as underweight (WAZ), or wasting (WHZ) were included separately in the models (data not presented).

Such characteristics between rotavirus diarrheas with non rotavirus diarrhea were observed for both the observation periods. In 1993–1997, significant association us observed with sanitary toilet use, antimicrobial use at home, vomiting, abdominal pain, watery stool, some or severe dehydration, intravenous fluid use, malnutrition (stunting) and co-pathogens (*Vibrio cholerae*, *Shigella* spp. and ETEC) (Table 2) in multivariate analysis.

**Table 2:** Changing socio-demographic, clinical and host characteristics, and co-infection among under-5 children between rotavirus and non rotavirus diarrhea in the period of 1993–1997.

Variables	1993–1997; rotavirus; n = 2,493 (%)	1993–1997; non rotavirus; n = 7,386 (%)	OR (95% CI)	aOR (95% CI)
Male	1538 (62)	4581 (62)	0.99 (0.90–1.08)	-
Maternal literacy	1312 (53)	3562 (48)	1.19 (1.09–1.31)*	0.88 (0.72–1.08)
Use sanitary latrine	1348 (54)	3578 (48)	1.25 (1.14–1.37)*	1.28 (1.05–1.57)*
Family size (>5 members)	954 (38)	2881 (39)	0.97 (0.88–1.07)	-
Slum residence	283 (11)	963 (13)	0.85 (0.74–0.99)*	0.91 (0.68–1.23)
Boiled drinking water	402 (16)	995 (13)	1.24 (1.09–1.40)*	0.96 (0.75–1.22)
Use of antimicrobial at home	1558 (63)	4358 (59)	1.16 (1.05–1.27)*	1.24 (1.03–1.49)*
History of measles within 6 months	225 (9)	678 (9)	0.98 (0.84–1.15)	-
Vomiting	2164 (87)	5318 (72)	2.56 (2.25–2.91)*	2.82 (2.21–3.60)*
Fever	262 (10)	810 (11)	0.95 (0.82–1.11)	-
Abdominal pain	491 (20)	1991 (27)	0.66 (0.59–0.74)*	0.81 (0.66–0.99)*
Duration of diarrhea (>24 hours)	1883 (76)	5718 (77)	0.90 (0.81–1.00)	-
Number of stool in last 24 hours (>10 times)	1058 (42)	3140 (42)	1.00 (0.91–1.09)	-
Watery stool	2408 (97)	6532 (88)	3.70 (2.93–4.68)*	3.41 (2.07–5.63)*
Some or severe dehydration	457 (18)	2069 (28)	0.58 (0.51–0.65)*	0.67 (0.51–0.89)*
Use of intravenous fluid	216 (9)	1490 (20)	0.38 (0.32–0.44)*	0.63 (0.44–0.89)*
Hospital stay ≥24 hours	1048 (43)	2941 (41)	1.09 (0.99–1.20)	-
Underweight (WAZ<−2)	1110 (45)	4396 (60)	0.54 (0.42–0.59)*	-
Stunted (HAZ<−2)	940 (38)	3962 (54)	0.51 (0.47–0.56)*	0.52 (0.43–0.63)*
Wasted (WHZ<−2)	611 (25)	2439 (34)	0.65 (0.58–0.72)*	-
*Vibrio cholerae*	94 (4)	895 (12)	0.28 (0.23–0.36)*	0.27 (0.19–0.39)*
*Shigella*	76 (3)	715 (10)	0.29 (0.23–0.38)*	0.39 (0.24–0.64)*
ETEC	112 (13)	299 (17)	0.71 (0.56–0.90)*	0.63 (0.49–0.81)*

On the other hand,) in multivariate analysis, such associations were observed, for maternal literacy, family size (>5), drinking boiled water, fever, stool frequency more than 10 times in last 24 hours, hospital stay (≥24 hours) in addition to other (at 1993–1997) during 2008–2012 except sanitary toilet use and, some or severe dehydration (Table 3).

**Table:** **Table 3.** Changing socio-demographic, clinical and host characteristics, and co-infection among under-5 children between rotavirus and non rotavirus diarrhea in the period of 2008–2012.

Variables	2008–2012; rotavirus; n = 2,493 (%)	2008–2012; non rotavirus; n = 3,711 (%)	OR (95% CI)	aOR (95% CI)
Male	1813 (63)	2276 (61)	1.09 (0.98–1.20)	-
Maternal literacy	2387 (83)	2904 (78)	1.39 (1.22–1.58)*	1.24 (1.07–1.44)*
Use sanitary latrine	2228 (78)	3025 (82)	0.79 (0.70–0.90)*	0.88 (0.76–1.01)
Family size (>5 members)	1018 (35)	1145 (31)	1.23 (1.11–1.37)*	1.15 (1.03–1.29)*
Slum residence	107 (4)	220 (6)	0.62 (0.48–0.78)*	0.79 (0.60–1.04)
Boiled drinking water	1078 (38)	1715 (46)	0.70 (0.63–0.78)*	0.75 (0.67–0.84)*
Use of antimicrobial at home	2339 (82)	2556 (69)	2.01 (1.78–2.26)*	1.60 (1.40–1.84)*
History of measles within 6 months	73 (2)	111 (3)	0.85 (0.62–1.16)	-
Vomiting	2125 (74)	2452 (66)	1.47 (1.32–1.64)*	1.52 (1.35–1.71)*
Fever	233 (8)	252 (7)	1.21 (1.01–1.47)*	1.30 (1.06–1.60)*
Abdominal pain	463 (16)	787 (21)	0.72 (0.63–0.81)*	0.77 (0.66–0.88)*
Duration of diarrhea (>24 hours)	2227 (78)	2623 (71)	1.45 (1.29–1.62)*	1.04 (0.91–1.19)
Number of stool in last 24 hours (>10 times)	1525 (53)	1663 (45)	1.40 (1.27–1.55)*	1.28 (1.14–1.43)*
Watery stool	2796 (98)	3512 (95)	2.31 (1.73–3.08)*	1.96 (1.44–2.65)*
Some or severe dehydration	985 (34)	1371 (37)	0.89 (0.81–0.99)*	0.99 (0.88–1.12)
Use of intravenous fluid	79 (3)	416 (11)	0.22 (0.17–0.29)*	0.25 (0.19–0.32)*
Hospital stay ≥24 hours	1196 (42)	1020 (28)	1.91 (1.71–2.12)*	2.20 (1.95–2.48)*
Underweight (WAZ<−2)	747 (26)	1265 (35)	0.67 (0.60–0.75)*	-
Stunted (HAZ<−2)	626 (22)	1055 (29)	0.69 (0.62–0.78)*	0.72 (0.63–0.81)*
Wasted (WHZ<−2)	530 (19)	856 (24)	0.74 (0.66–0.84)*	-
*Vibrio cholerae*	32 (1)	369 (10)	0.10 (0.07–0.15)*	0.15 (0.10–0.22)*
*Shigella*	21 (1)	186 (5)	0.14 (0.09–0.22)*	0.17 (0.10–0.27)*
ETEC	193 (7)	441 (12)	0.54 (0.45–0.64)*	0.57 (0.47–0.69)*

## Discussion

One of the major observations of the present study was the increasing trend of rotavirus diarrhea cases among under-5 children which is an alarming public health concern and reiterates the demand of mass vaccination. This might be a parallel effect of increasing diarrhea patients in the hospital; however, Das SK et. al. reported the changing patient population in the Dhaka Hospital of icddr,b with increasing trend among adult and elderly [17]. Over the period, the proportion of bacterial infection such as *Vibrio cholerae, Shigella* spp. and ETEC have been reduced which might be explained the increased rotavirus diarrhea [17]. Another interesting observation was association of rotavirus diarrhea with the nutritional status of the children. For example, higher proportion of well-nourished children during 2^nd^ observation period (2008–2012) had rotavirus diarrhea which was found to be higher among malnourished children during 1^st^ observation period (1993–1997). Usually, rotavirus diarrhea is considered as a disease of well nourished children due to higher presence of receptors for rotavirus at binding site in healthy lining epithelium of intestinal mucosa [18]. On the other hand, in malnourished children there are pathological changes of intestinal mucosa due to several micronutrient deficiencies, compromised immunity and repeated infections resulting in destruction of binding sites [19]. Over the period, as there is significant reduction of childhood malnutrition which might explain our present observation of increased frequency of rotavirus cases due to reduced malnourished children presenting with diarrhea; although, achievement in substantial reduction of childhood malnutrition to reach MGD 4 still lagging behind [20].

The present study also noted several changes, specially socio-demographic and clinical indicators among under-5 rotavirus diarrhea children between two observation periods. For example; increase in maternal literacy, more use of sanitary toilets, higher boiled water drinking, frequent use of antimicrobial at home, and lesser slum dwelling. These might be due to overall gross national improvements in maternal level of education; and health and hygiene practices[21]. All these indicators had significant association with childhood diarrhea as reported elsewhere [22].

On the other hand, clinical presentations such as frequent vomiting, and higher frequency of stool (>10 times/24 hours) might contribute to increased proportion of dehydrating diarrhea. Interestingly, use of intravenous saline for initial correction of dehydration significantly decreased which implies optimal acceptance of the use of ORS at home soon after the onset of diarrhea [23]. These, once again are related with increased literacy as well as acquiring related health education from mass media [24], [25]. However, increased use of antimicrobial at home for rotavirus diarrhea is an alarming public health challenge as antimicrobials have no role at all in rotavirus diarrhea [26].

The observation of decreasing trend of co-infection of rotavirus with *Vibrio cholerae, Shigella* spp. and ETEC from 1^st^ to 2^nd^ observed period might be due to the relation with compromised immunity from malnutrition as well as history of measles within 6 months prior admission in 1^st^ observation period [27].

## Limitations

Although unbiased systematic sampling method was used to enroll patients into surveillance system irrespective of age, sex, nutritional status, disease severity or socioeconomic background and large dataset with standard laboratory facility were supportive of the strengths of this analysis. There might be a sampling bias by enrolling every 50^th^ patient and hospital data might not be the representative of general population.

## Conclusion

The present analysis clearly indicated increasing trend of rotavirus diarrhea among under-5 children in urban Bangladesh. This observation was also correlated with several socio-demographic, clinical and host characteristics which changed significantly over the period especially water sanitation, hygiene practices, and improved nutritional status of the child. Thus, vaccination against rotavirus may be prioritized by the policy maker with improved water-hygiene practices among this high risk group.
